# Characteristics of tiger moth (Erebidae: Arctiinae) anti-bat sounds can be predicted from tymbal morphology

**DOI:** 10.1186/s12983-019-0345-6

**Published:** 2019-12-10

**Authors:** Nicolas J. Dowdy, William E. Conner

**Affiliations:** 1 Department of Biology, Wake Forest University, Winston-Salem, North Carolina USA; 2 Invertebrate Zoology, Milwaukee Public Museum, 800 W. Wells Street, Milwaukee, WI USA

**Keywords:** Bioacoustics, Lepidoptera, Anti-predator defense, Predictive modeling, Collections-based research

## Abstract

**Background:**

Acoustic signals are used by many animals to transmit information. Variation in the acoustic characteristics of these signals often covaries with morphology and can relay information about an individual’s fitness, sex, species, and/or other characteristics important for both mating and defense. Tiger moths (Lepidoptera: Erebidae: Arctiinae) use modified cuticular plates called “tymbal organs” to produce ultrasonic clicks which can aposematically signal their toxicity, mimic the signals of other species, or, in some cases, disrupt bat echolocation. The morphology of the tymbal organs and the sounds they produce vary greatly between species, but it is unclear how the variation in morphology gives rise to the variation in acoustic characteristics. This is the first study to determine how the morphological features of tymbals can predict the acoustic characteristics of the signals they produce.

**Results:**

We show that the number of striations on the tymbal surface (historically known as “microtymbals”) and, to a lesser extent, the ratio of the projected surface area of the tymbal to that of the thorax have a strong, positive correlation with the number of clicks a moth produces per unit time. We also found that some clades have significantly different regression coefficients, and thus the relationship between microtymbals and click rate is also dependent on the shared ancestry of different species.

**Conclusions:**

Our predictive model allows the click rates of moths to be estimated using preserved material (e.g., from museums) in cases where live specimens are unavailable. This has the potential to greatly accelerate our understanding of the distribution of sound production and acoustic anti-bat strategies employed by tiger moths. Such knowledge will generate new insights into the evolutionary history of tiger moth anti-predator defenses on a global scale.

## Background

Acoustic signals are used by many animals to transmit information to receivers. Variation in the acoustic characteristics of these signals can aid in species discrimination and the assessment of potential mates [1]. Acoustic variation can also signal information about traits related to fitness such as body size [2]. Beyond the communication of information, the acoustic qualities of such sounds can also affect their physical transmission through the environment [3]. Variation in the acoustic characteristics of these signals is well-known to covary with the morphology of the sound-emitting organ in a variety of animal groups (Aves: [4, 5]; Pisces: [6]; Anurans: [7]; Mammals: [8]; Insects: [9]).

**Fig. 1:**
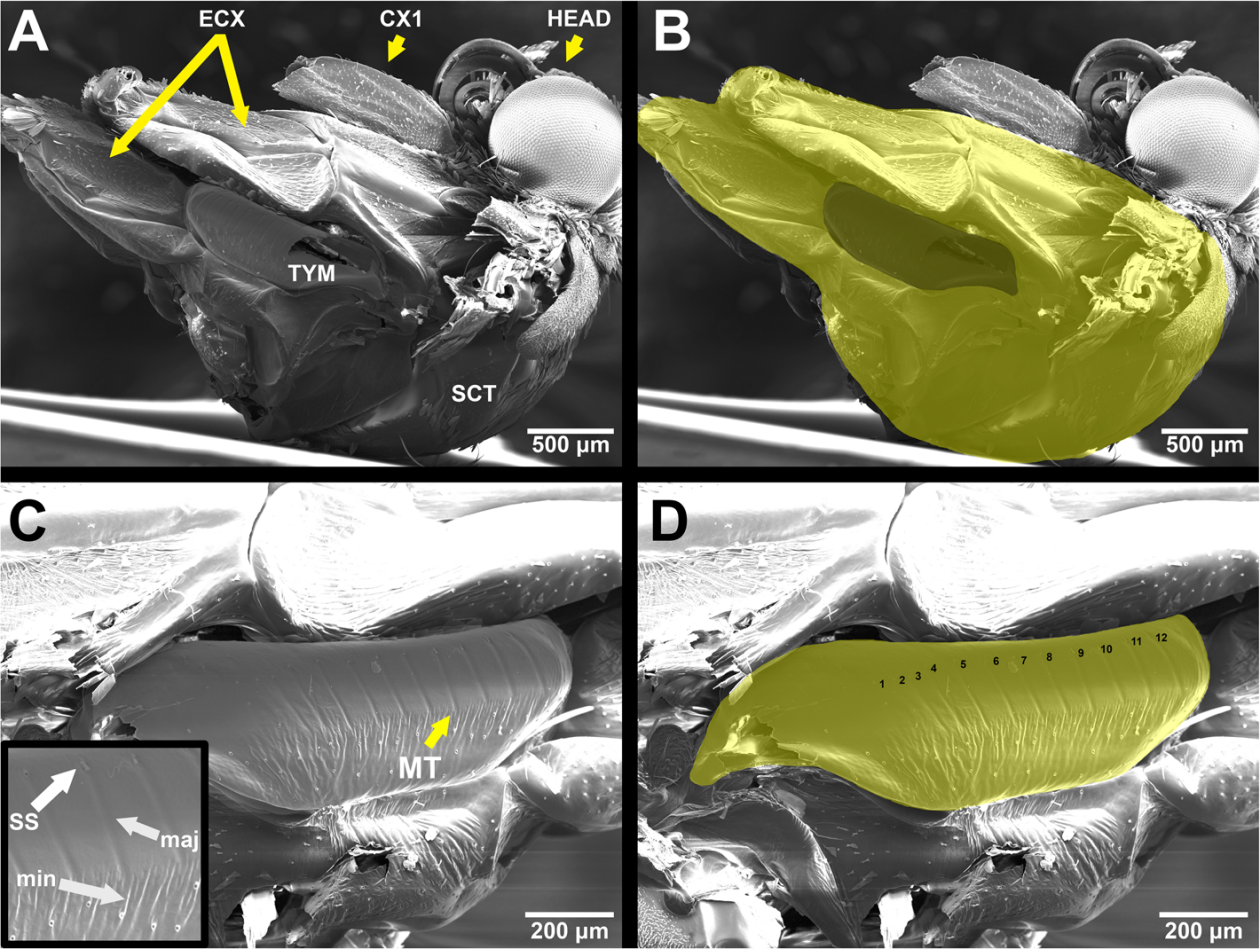
Scanning electron micrographs of *Cisthene martini*. **a** Thorax with tymbal; ECX: eucoxae of meso- and metathorax, CX1: coxa of prothorax, TYM: tymbal, SCT: scutum, HEAD: head. **b** Projected surface areas of the thorax (THSA) and tymbal (TYSA) highlighted in lighter and darker yellow regions, respectively. **c** Magnified image of tymbal; inset: further magnified view of microtymbals (MT), ss: scale sockets, maj: major grooves, min: minor grooves. **d** Magnified image of tymbal highlighted in yellow with microtymbals numbered in order from anterior to posterior. Scale bars in each image. **a–d** are oriented with the dorsal side towards the bottom and the ventral side towards the top. **a**, **b** are oriented with the anterior side towards the right and the posterior towards the left. **c**, **d** are oriented with the posterior towards the right and the anterior towards the left

Tiger moths (Lepidoptera: Erebidae: Arctiinae) produce clicking sounds in response to the echolocation of bat predators and, in some species, during courtship [10–14]. Trains of clicks are generated by the cyclical buckling of a specialized pair of metathoracic tymbal organs whose surfaces are marked by corrugations called microtymbals organized along a “striated band” (Fig. 1c [10, 15];). Depending on the species, the sounds produced by tiger moths can vary greatly in frequency, intensity, the number of clicks produced per flexion and relaxation of the tymbal, and other characteristics [16, 17]. When these sounds are produced in a defensive context against bats, they serve to signal the unprofitability of the moth (i.e., acoustic aposematism), to mimic the aposematic signals of other tiger moths species (i.e., Batesian or Müllerian acoustic mimicry), and/or to disrupt the echolocation cries of bats in an attempt to escape predation (i.e., sonar jamming) [19–21]. It has been suggested that moth clicks can also startle bat predators. However, laboratory evidence has shown that bats habituate to moth clicks quickly, and therefore startle is not expected to be a major function in a natural context where the encounter rate with tiger moths is likely high [22, 23]. Though the mechanism and functions of sound production in tiger moths is well-understood, the physical properties of the tymbal organ that give rise to the variation in their sonic characteristics remain under-studied.

Tiger moths are one of the most diverse subfamilies of Lepidoptera, with more than 11,000 species currently described [24–26]. Acoustic traits of most of these species are still uncharacterized, impeding our understanding of the evolution of acoustic behaviors within the group. We believe it is important to determine whether and how precisely aspects of sound production can be predicted from the morphological analysis of tiger moth tymbal organs, as this would allow for a broad preliminary assessment of sound production for this diverse group on a global scale. Additionally, inferences of sound production could be made for species rarely encountered in nature but preserved in natural history collections (e.g., pinned, papered, or ethanol-preserved material), species which have recently gone extinct, or perhaps even for fossilized material with at least one intact tymbal organ [27].

We present here the first study to examine how the morphology of the tymbal organ determines the maximum number of clicks a moth produces per second (i.e., “maximum click rate”; CR). To accomplish this, we recorded the anti-bat acoustic responses from wild-caught tiger moths and simultaneously examined the major morphological features of the tymbal organs of these recorded moths. These morphological characters included the ratio of projected tymbal and thorax surface area (T2T) and the number of striations on its surface (i.e., “microtymbals”; MT). Tiger moth tymbals share some similarities with those of cicadas, which possess “tymbal ribs” that act as buckling points which produce individual clicks [28–31]. Each MT is thought to contribute a single click during the activation of the tymbal organ, we hypothesized that MT would have a strong effect on CR. We have chosen to model CR because it has been hypothesized to strongly influence the anti-bat function of these sounds. It has been suggested that the sonar-jamming strategy of tiger moths could be subject to a “duty-cycle threshold” ([17]; also see Kawahara and Barber, 2015 for Sphingidae). When moth clicks are produced at a rate above this threshold value, they are hypothesized to occur with sufficient frequency to reliably produce a disruptive effect. When clicks are produced at a rate below this threshold, they are hypothesized to occur too infrequently to reliably produce a jamming effect, instead functioning as acoustic aposematic or mimetic signals. These results represent the first step towards predicting the acoustic characteristics and possibly the defensive functions of the anti-bat sounds of tiger moths on a broad scale.

## Results

### Tymbal morphology and acoustic measurements

Data collected in this study are given in Additional file 1. We examined the tymbal morphology and anti-bat sounds produced by 70 individuals from 69 species, 38 genera, and 7 higher taxonomic groupings (i.e., CLADE) of Arctiinae. In nearly all cases, we examined one specimen per species, except for *Amaxia juvenis*, which included two individuals. The distribution and descriptive statistics of CR, MT, and T2T are given in Additional file 2.

### Predicting click rate from Tymbal morphology

**Fig. 2:**
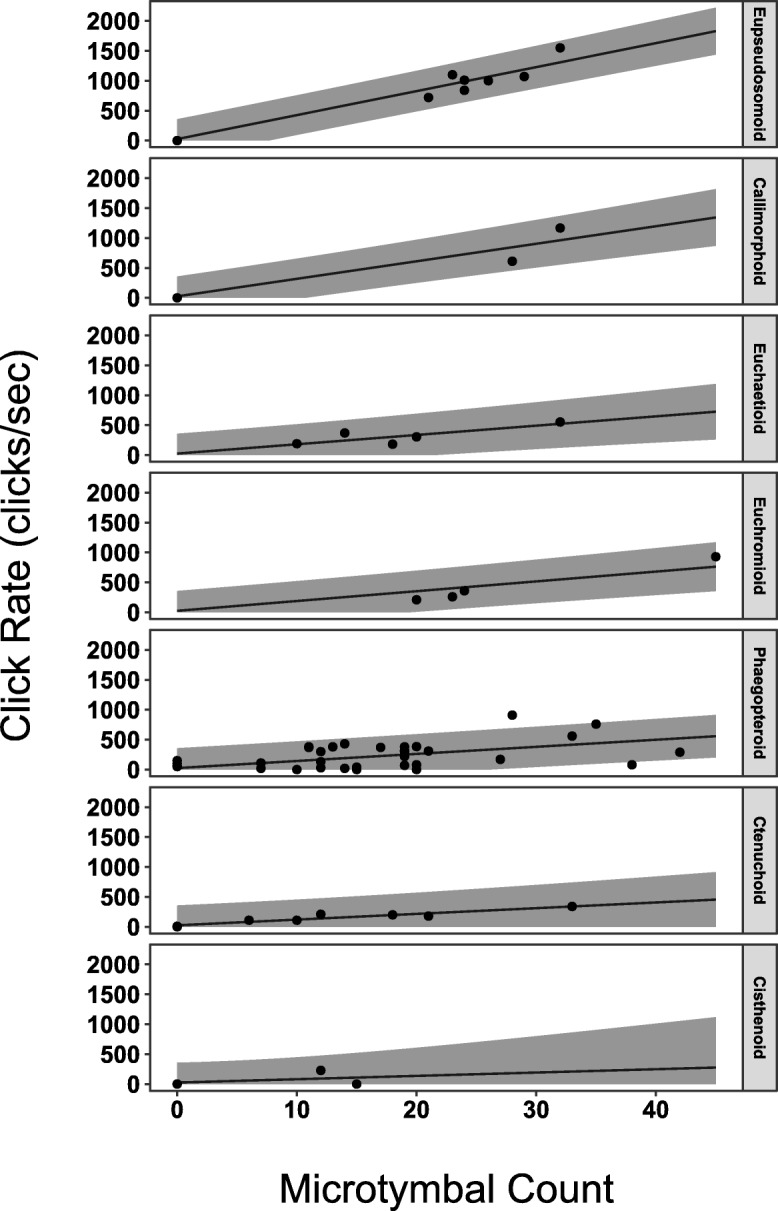
Model 9 with 95% Prediction Intervals. Given MT and CLADE measured from a specimen, its CR is expected to fall within these intervals in 95% of cases. CR which were predicted to be negative values (e.g., - 100 clicks/second) were set to 0 clicks/sec because negative rates would not be biologically meaningful

**Fig. 3:**
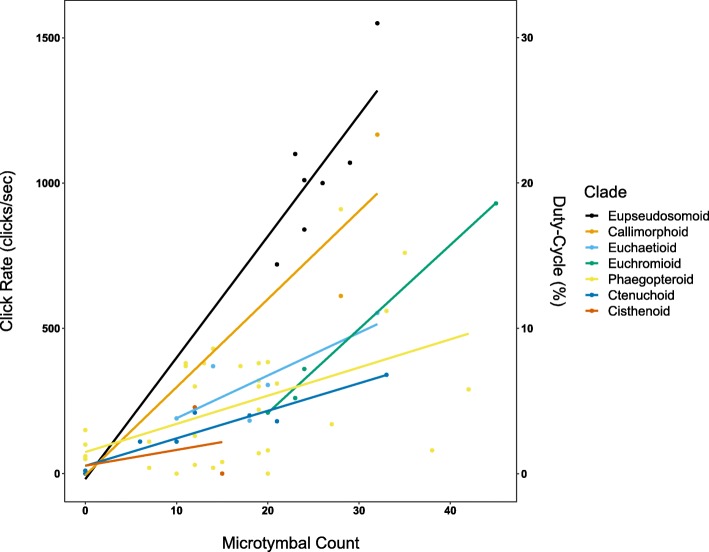
Plot of MT against CR by CLADE. Most clades exhibit a positive relationship between microtymbal count and click rate, but to varying degrees. Eupseudosomoids were found to have the steepest, positive slope, followed by Callimorphoids. The Cisthenoid lineage was the only clade not found to have a slope significantly different from zero. Among the remaining clades, the relationship was positive, significantly greater than zero, and largely consistent

We found two strongly supported models predicting CR from MT, T2T, and CLADE (Additional file 3). Both models explain a large proportion of the variation in CR (Adj. *R*^*2*^ = 0.79). The predictor coefficients and adjusted R^2^ are similar for both models. Though the more complex “Model 11” has somewhat lower RMSE compared to “Model 9”, this was not a significant difference at the standard cutoff (ANOVA: F = 3.4, *p* = 0.07). We built prediction intervals for each model indicating where CR is predicted to lie in 95% of cases for a given MT, CLADE, and T2T (Model 9: Fig. 2; Model 11: Additional file 4). Both models support an intercept that is not significantly different from 0. The results from Model 9 suggest that the slope of the relationship between MT and CR for most CLADEs was significantly different from 0. The Cisthenoid clade slope was positive, but not significantly greater than 0. However, the sample size for this clade was very low (*n* = 3). We constructed a set of CLADE level contrasts and applied them to Model 9 and Model 11 to compare the results among all CLADEs (Additional file 5; Additional file 6). We found that the Eupseudosomoid and Callimorphoid clade had significantly greater slopes than all other examined clades, suggesting that they produce higher CR for a given number of MT (Fig. 3). Some species lacking microtymbals are still capable of producing sound (*n* = 5, 7%) and some species with microtymbals did not produce sound in our trials (*n* = 4, 6%) (Additional file 1). Nonetheless, both models were capable of robustly predicting CR for all but one clade.

## Discussion

In both of our models, MT and CLADE were critical and significant factors for predicting CR. In Model 11, T2T also played a significant, albeit weaker role when compared to MT and CLADE. However, Model 11 and Model 9 accounted for a similar proportion of variance in CR. We prefer Model 9 as it requires measuring only a single aspect of tymbal morphology (i.e., MT) whereas Model 11 requires three (i.e., MT, TYSA, THSA) without a significant improvement in CR prediction.

The Eupseudosomoid clade contains the only tiger moth species currently known to jam bat sonar based on empirical evidence [32]. Interestingly, this group was found to have a significantly steeper slope relating CR to MT than all other clades (Fig. 3). The Callimorphoid clade also exhibits a significantly steeper and positive relationship between CR and MT relative to all other clades, though to a lesser degree than the Eupseudosomoids. Members of the Callimorphoids have never been assessed for sonar jamming. Species in this study that are known to possess aposematic or mimetic anti-bat functions based on empirical data were members of CLADEs found to have shallow slopes (i.e., Euchaetioid: *Pygarctia roseicapitis* [21], *Cycnia tenera*, *Euchaetes egle* [19]; Cisthenoid: *Cisthene martini* [21]). We believe the Eupseudosomoids and Callimorphoids may have evolved other mechanisms to increase CR, in addition to increasing MT. These could include behaviors such as a higher degree of asynchrony between contralateral and ipsilateral tymbal activation, relative timing of tymbal activation, more complete utilization of the microtymbals along the striated band, and/or other factors. Whatever the mechanisms, we believe these two clades are likely to contain a greater proportion of species capable of a sonar jamming function that is reliant on high CR. Among all the clades included in this study, only the Cisthenoid lineage was found to have a slope not significantly different from 0. We believe the most likely explanation is our low sample size (*n* = 3) within this clade.

It is not yet clear whether the CR of species bearing no microtymbals can be predicted from morphology alone. While most species without microtymbals did not produce sound in our trials, some species did, generally producing just a single click as the entire tymbal surface buckles. It is not currently clear what distinguishes these two groups, but this should be explored further with more data. Likewise, it would be useful to determine whether any factors can explain why a small number of species with microtymbals did not produce sound in our trials. It is possible that these observations simply represent false negative responses to simulated bat echolocation. It is also possible that these species only use their tymbals in other contexts, such as in intraspecific communication during courtship [14], and so we emphasize that the interpretation of reports indicating a lack of sound production among tiger moths should bear in mind the context and methods used to elicit those responses, the sex, and the number of individuals used to make that determination. However, we note that many unresponsive individuals with a non-zero number of microtymbals possessed very shallow, irregularly spaced, and/or irregularly shaped microtymbals, suggestive of a potentially low- or non-functioning vestigial state (e.g., Additional file 7).

The results of this study are a significant first step toward predicting the click rates of tiger moth species from morphology alone, and perhaps even the function of those sounds. Jamming thresholds have been measured using both maximum duty-cycle (DC) ([17]; Kawahara and Barber, 2015) and CR (Fernandez et al., unpublished). We prefer CR because it does not rely on measuring click duration which is affected by recording and analysis methodologies (e.g., the intensity of the signals and the arbitrary definition of the start/end of signals). By combining predicted CR and a CR threshold for sonar jamming we can construct hypotheses assigning species to acoustic anti-bat strategies (i.e., aposematic/mimetic signaling versus sonar jamming). This would aid us in understanding the evolutionary patterns of sound production in tiger moths (e.g., have multiple lineages of tiger moths independently converged on a sonar jamming anti-bat strategy?).

The models presented in this study should be viewed as well-supported hypotheses, but their predictive powers should be verified using an independent dataset of moth sounds and tymbal morphology. While our models explain a large amount of variation in CR they could be further improved by adding data from more species, particularly from genera that have not yet been included. Data from individuals within clades that were not represented in this analysis (e.g., the subtribes Lithosiina and Nudariina) should be added so that CR can be also predicted for members of those clades. In addition, while CLADE captures some of the statistical dependence among data due to phylogenetic relatedness, a statistically robust phylogeny would allow us to better account for this. We could also incorporate additional predictor variables to better understand and account for the underlying sources of variation in CR. Furthermore, this approach could easily be applied to other aspects of tiger moth sounds. For example, this approach could be used to study which tymbal features account for the high degree of variation in the dominant frequency, intensity, or duration of clicks observed between species [32].

Among Lepidoptera, the Arctiinae are not alone in utilizing tymbals for sound production. A number of major lepidopteran lineages have convergently evolved tymbal or tymbal-like organs in order to produce sounds for courtship or defense (e.g., Geometridae [33]; Nolidae [34]; Lymantriinae [35]; Noctuidae [36]; Pyralidae [37]; Crambidae [38]; for an overview, see [39]). Similar structures can also be found in other insect lineages, including cicadas and other subgroups of Hemipterans [28–31, 40]. It is likely that the methods from this study will be generally applicable to other tymbal-bearing insect groups since many tymbals share the same basic structure and function as those of the Arctiinae.

## Conclusion

Natural history collections are invaluable sources of data for disciplines as diverse as biogeography, ecology, genetics, and systematics. Yet even recent reviews of their potential usefulness overlook their possible applications to the study of behavior [41–43]. We believe this study is a good example of how even preserved specimens can provide useful information about the behaviors these specimens may have exhibited in life. We expect that investigations of other animal behaviors could also benefit from collections-based research to lead to insights about the diversity and distribution of behaviors on large spatial or temporal scales.

The predictive models presented here cannot replace the direct measurement of anti-bat sounds. However, by leveraging natural history collections this method can give us insights into the acoustic behaviors of species which are rarely encountered, those which have recently gone extinct, or perhaps even from fossilized material [27, 44]. In addition, while tiger moths occur worldwide, their diversity is highest in tropical regions where the probability of significant biodiversity loss is very high [45]. As deforestation, global climate change, and other sources of biodiversity loss continue largely unabated, it is possible that the sounds of some species could be lost to science [46]. We believe our model can be used to great effect as a complement to direct measurements of sound production in order to quickly and broadly expand our understanding of the acoustic characteristics of tiger moth sounds and the anti-predator strategies they employ.

## Methods

### Field site and insect capture method

Field experiments were mainly conducted at the Yanayacu Biological Station and Center for Creative Studies (YBS) approximately 5 km west of Cosanga, Ecuador (00°36.235′ S, 77°52.917′ W; elevation: 2100 m), between August 21st – 29th, 2013. This location was chosen based on the impressive amount of information available regarding the many moth species present there [47]. YBS lies on the eastern slopes of the Andes and is comprised of primary forest as well as partially reforested pastures and roadsides. A subset of our data included moths from field sites in Arizona, North Carolina, Texas, and Michigan. This was done to extend our analysis to a broader geographic range and to include measurements of some of the classic, well-studied species from previous research efforts. Arizonan moths included in this study were captured throughout July 2013 at the Southwestern Research Station (SWRS) operated by the American Museum of Natural History (31.883985°, - 109.206064°; elevation: 1650 m). North Carolinian moths were captured on private property with permission of the property owner on July 23rd, 2015 at a location approximately 4.5 km north west of Elk Knob State Park (36.332629°, - 81.695645°; elev.: 1350 m). Moths were captured in Michigan on private property with permission of the property owner in July 2014 at a location approximately 8.5 km north-north west of Lapeer, Michigan (43.051434°, - 83.318839°; elev.: 262 m). Texan moths were captured near Liberty, TX in May 2014 (30.098340, - 94.765879; elev. 13 m). All insects were collected from sheets illuminated with 15 W ultraviolet “quantum” lights (Leptraps.com; F15T8QBL) and placed individually in 30 mL plastic containers and stored for up to 24 h at ambient outdoor temperatures (12–15 °C) prior to acoustic recordings.

### Acoustic recordings

Freshly captured moths were held by the wings, which were folded above the thorax and restrained with a hemostatic clamp. All recordings were made in a darkened room at night in ambient outdoor temperatures (12–15 °C). An Avisoft Bioacoustics USGH digital recording unit was connected to a single Avisoft CM16/CMPA ultrasonic microphone (± 3 dB from 15 to 140 kHz) and set to record at a sampling rate of 250–500 kHz. The microphone was placed perpendicular to the midline of the moth body, 10 cm from the thorax of the individual (where the sound-producing organs are located). We included clicks from both the ipsilateral and contralateral tymbals (identifiable based on their relative intensities) in our analysis. An AT100 ultrasonic speaker (Binary Acoustic Technology) was placed 10 cm from the posterior end of the moth thorax where the tympanal hearing organs are located, and parallel to the midline of the body. Moths were stimulated to produce sound by playing a pre-recorded echolocation attack sequence from the insectivorous big brown bat, *Eptesicus fuscus* (Chiroptera: Vespertilionidae). This species of bat was chosen because it is one of the few bat species sympatric with all moth species included in this study [48, 49]. The search, approach, and buzz phases of bat echolocation were all present and spanned a pulse interval of 115 ms in search phase to 6 ms in the buzz phase. Echolocation intensity reached and then sustained a peak equivalent Sound Pressure Level of 100 dB at 10 cm in the approach phase. For more details see previously reported methods [16]. Stimuli were repeated seven times per individual with approximately 4–5 s of silence between trials. Files were saved in a. WAV format. Each recording contained only a single simulated bat attack.

### Specimen vouchering

After acoustic assays were completed, each specimen was euthanized in a freezer (- 20 °C) for 24 h. Afterwards, the specimens were thawed and then field pinned. Each specimen was pinned on top of an 18% grey card and the wings were spread and pinned in place with insect pins. A metric photographic scale (1 mm increments) was placed next to each specimen. We photographed the dorsal and ventral sides of each specimen using a Canon XTi DSLR (10.1 MP; RAW image format; shutter speed: 1/250 s) with Canon EF-S 60 mm Macro Lens (manual aperture of f/11) and a Canon MT-24EX Macro Twin Lite Flash for illumination. Once photographs were taken, we removed the legs, antennae, proboscis, abdomen, and wings and placed each into separate 1.5 mL tubes filled with 95% EtOH or glassine envelopes. The thorax and head were then placed into their own 1.5 mL tube filled with 95% EtOH. All tissues were stored at - 80 °C and are currently archived at the Milwaukee Public Museum.

### Scanning Electron microscopy

We used a scanning electron microscope (SEM) (Model: Amray 1810) to image the tymbal organs. To prepare the specimens for imaging we removed each thorax from its 1.5 mL tube and evaporated the EtOH by air drying for 15–30 min under a fan. We found that critical point drying was not necessary for these specimens. To make the tymbal and microtymbals more clearly visible and easily countable we used a combination of compressed air, scotch tape, and forceps to remove the scales from the surface of the tymbal and thorax, taking care not to damage or puncture the tymbal surface. In some cases, we also removed the mesepisternum and/or mesepimeron to make imaging the anterior edge of the tymbal easier. The specimens were placed on stubs with double-sided carbon tape and were gold coated in a sputter coater (Model: Cressington Scientific Sputter Coater 108) for 30 s under argon gas. Images were taken using an acceleration potential of 10–12 kV and saved as. TIF. Only a single side of each specimen was imaged. One image was taken as a direct side-on view of the body such that both the thorax and tymbal organ could be seen. A second image of the tymbal was taken at higher magnification to facilitate the counting of the microtymbals (Fig. 1a, c).

### Image analysis

Images from SEM were analyzed in Adobe Photoshop CC (Adobe Systems, San Jose, California). First, two separate layers were created for the tymbal and the thorax using the lower magnification SEM image. The tymbal and thorax were outlined in their respective layers using the Paintbrush Tool and filled in (Fig. 1b, d). Our thorax measurements include the eucoxae of the meso- and metathoracic segments as well as the entirety of the scutum (Fig. 1a). We excluded the coxa of the first thoracic segment and the patagia because these parts were sometimes missing or damaged and so could not be measured for every specimen. The Ruler Tool was used to set the scale between pixel and millimeters using the scale bar embedded in each image from the SEM image capture software. Each layer was selected using the Magic Wand Tool and the Record Measurements button yielded the projected surface area measures for the tymbal and thorax. The second SEM (zoomed) image was used to count the number of visible microtymbals. We discovered that at least two types of microtymbal can be present. The first class we dub “major grooves” (Fig. 1c, inset, “maj”) which are depressions in the surface of the tymbal along the tymbal surface (collectively referred to as the “striated band” [15];) which are usually accompanied by a singular scale socket (Fig. 1c, inset, “ss”) and correspond best with the traditional definition of a microtymbal. The second class we dub “minor grooves” (Fig. 1c, inset, “min”) which resemble a wrinkling of the tymbal surface and can occur between major grooves or even along the posterior edge of the striated band. We disregarded the minor grooves in this analysis as it is not clear whether the minor grooves contribute to sound production. Future investigations of the functional morphology of tiger moth tymbals should examine whether these structures have a function, and if so, what that function is. We counted the absence of microtymbals as 0 microtymbals.

### Acoustic analysis

#### Click detection and measurement

We used Avisoft SASLab Pro (Avisoft Bioacoustics, Berlin, Germany) to detect and measure the number of moth clicks present in each of our recordings. For each. WAV file we generated a spectrogram with the following parameters: FFT length = 256, Frame Size = 50%, Window = FlatTop with a window overlap of 96.87% (8 samples). We then used the Automatic Parameter Measurements tool to automatically identify the moth clicks in our files. To do this, we used a two-threshold approach. The threshold defining when a signal should be classified was variable depending on the intensity of the individual moth. The threshold defining the end of a detected signal was -8 dB relative to the peak intensity of that signal. After processing each file with the automatic method, we manually went through and removed spurious results, manually included clicks that were not detected, and manually separated individual clicks when multiple clicks occurred too close together in time and were classified as the same signal. The timestamps of each click were saved into a. CSV file for further analysis.

#### Measuring maximum click rate

This study uses the maximum click rate produced by a given moth as a measure of the rate of its sound production, which we refer to as “CR”. This was chosen because it is less sensitive to incomplete activations of the tymbal organ that may result from our recording methodology. CR is defined as the largest number of clicks present in any given 100 ms time window. This value is multiplied by 10 and reported in terms of the number of clicks that would be produced per 1 s. To measure CR, we wrote a custom R script which took as its input the. CSV files generated in SASLab Pro. This script starts from the first detected click in a recording and counts the number of detected clicks that occur within 100 ms. In further iterations, this 100 ms time window is shifted by a single click event and the click rate within the new window is calculated. Once the window reaches the final click in a recording the maximum recorded click rate among all windows is determined and reported for a given recording. CR measurements from multiple simulated bat attacks against the same moth are then compared and the overall maximum is retained and reported for that individual.

### Linear regression model selection

#### Model selection

We measured three aspects of tymbal morphology: (1) the number of microtymbals (MT), (2) the projected tymbal surface area (TYSA) expressed in mm^2^, and (3) the projected thorax surface area (THSA) expressed in mm^2^. We also calculated and included (4) the ratio of projected tymbal surface area to projected thorax surface area (T2T). We examined a correlation matrix between these variables to determine which should be included in our model (Additional file 8). We chose to retain MT and T2T in our final models. MT was retained because of its large correlation with CR and to examine whether CR can be predicted from MT. T2T was retained for its positive correlation with CR, but also because it contained information about both TYSA and THSA. While the correlation coefficients of TYSA and T2T with CR were similar, we chose to retain only T2T as this number more strongly correlated with MT while also incorporating TYSA and THSA.

Unfortunately, we currently lack a robust phylogeny of Arctiinae for use in comparative analyses designed to account for the phylogenetic nonindependence between data points. In these cases, it is preferred to use taxonomy to account for at least some amount of shared ancestry [50]. Recent advances in our understanding of tiger moth relationships allow many species to be grouped into certain monophyletic groups [51, 52]. We defined the clades for our specimens from these studies (Additional file 9). Members of each clade are relatively similar morphologically and, along with the known phylogenetic relationships, classification of species into these clades is not difficult in most cases. We incorporated clade membership (CLADE) into our linear models to control for the phylogenetic nonindependence of our data [53–56].

To assess the phylogenetic dependence between data to the extent currently possible, we examined the linear relationships between each predictor by CLADE. We found that the relationship between CR and MT was positive within CLADEs, but the slope and possibly the intercept of the relationship may differ between CLADEs (Fig. 3). This prompted us to include models with an interaction term between MT and CLADE. The relationship between CR and T2T within CLADE was less clear, with some CLADEs exhibiting a positive correlation, some negative, and others with no discernable relationship at all (Additional file 10). However, because the relationships between CR and T2T did vary depending on CLADE, we also included models containing an interaction term between CLADE and T2T.

Our final model set included 19 models. We used Akaike’s Information Criterion corrected for small sample size (AICc) to rank and select the best model as implemented in the aictab function of the AICcmodavg package in R [57, 58]. Models less than 2 *∆* AICc units from the “top model” (lowest AICc value) were considered to be of similar support, while models greater than 9–11 ΔAICc units from the top model were considered to have relatively low support [59]. The results of AICc model ranking returned two top models of differing complexity from which we infer our results (Additional file 3).

#### Checking model assumptions

We determined our final model met the assumptions of linear regression by confirming the mean of the residuals was equal to zero, by visually checking for homoscedasticity of the residuals and normality using the plot command in base R, checking for the absence of autocorrelation with Durbin-Watson test implemented from the lawtest package in R (DW = 1.87, *p* = 0.24), and by ensuring that the residuals were uncorrelated with the predictors using cor.test function from base R [60, 61]. Tables were prepared in LaTeX using the xtable and texreg libraries within R [62, 63].

## Supplementary information

**Additional file 1.** Acoustic and morphological measurements. The recorded maximum click rates (CR), microtymbal counts (MT), projected tymbal surface area (TYSA; mm^2^), projected thorax surface area (THSA; mm^2^), and ratio of TYSA to THSA (T2T) are given for each individual included in the study. The higher taxonomic grouping each belongs to (CLADE), sex, and voucher identification numbers (id) are also given for each specimen. Species identifications were left at “sp” when definitive species level identifications required examination of gentilic morphology. The “cf” designation was used to indicate a close external similarity to a given species, but a definitive identification could not be made due to small deviations in external morphology.

**Additional file 2:** Descriptive statistics and distributions of CR, MT, and T2T. Individual data points from the 70 individuals included in our analyses are plotted, along with information about their distributions and summary statistics.

**Additional file 3: **AICc Model Comparisons. The relative performance of all examined models are given with their Corrected Akaike’s Information Criterion (AICc), ordered from strongest likelihood to weakest likelihood. Models within a *∆* AICc of 2 relative to the most likely model (i.e., M11) are considered to be equally supported.

**Additional file 4:** Model 11 with 95% Prediction Intervals. For a given MT, CLADE and T2T, CR is expected to fall within these intervals in 95% of cases. T2T shifts this prediction interval up or down depending on its value. In order to present the prediction intervals for Model 11 in a 2D graphic, we plotted two ribbons which represent the minimum (1.8%; dark grey) and maximum (16.7%; light grey) T2T values observed in this study. This shows the extent that the prediction interval could be expected to shift if two individuals within the same CLADE had the same MT, but extremely different T2T. CR which were predicted to be negative values (e.g., − 100 clicks/second) were set to 0 clicks/sec because negative rates would not be biologically meaningful.

**Additional file 5:** Contrast matrix for Model 9. The modeled relationship between click rate and microtymbal count clusters into 3 significantly different groups: Eupseudosomoids, Callimorphoids, and all other clades. Apart from the Cisthenoid clade, all clades were found to have a significantly positive relationship between microtymbal count and click rate. The significance and the magnitude of the slope differences are relative to the unlisted clade for each contrast column. Overall, Model 9 accounts for CR well (Adj. R^2^ = 0.79), while only requiring 2 factors to be measured (i.e., MT and CLADE).

**Additional file 6: **Contrast matrix for Model 11. The modeled relationship between click rate and microtymbal count clusters into 3 significantly different groups: Eupseudosomoids, Callimorphoids, and all other clades. Apart from the Cisthenoid clade, all clades were found to have a significantly positive relationship between microtymbal count and click rate. The significance and the magnitude of the slope differences are relative to the unlisted clade for each contrast column. Overall, Model 11 accounts for CR as well as Model 9 (both Adj. *R*^*2*^ = 0.79), while requiring 3 factors to be measured (i.e., MT, CLADE, and T2T).

**Additional file 7: **Comparison of normal and putatively vestigial microtymbal morphology. Exemplar microtymbal (MT) morphology of two species from closely related genera within the same CLADE (Phaegopteroid). A) tymbal of *Leucanopsis* cf *falacra* (index: 28; id: YAN13_0114), scale bar = 200 μm; inset: dorsal view of specimen, scale bar = 1 cm. B) tymbal of *Elysius deceptura* (index: 66; id: YAN13_0157), scale bar = 200 μm; inset: dorsal view of specimen, scale bar = 1 cm. C) represents a normal state (MT = 19; CR = 300) with regularly spaced, deep, and well-aligned microtymbals. D) represents a putatively vestigial state (MT = 10; CR = 0) with irregularly spaced, shallow, and misaligned microtymbals. A-C are oriented with the anterior side towards the left, posterior towards the right, dorsal towards the top, and ventral towards the bottom. D is oriented looking down the row of microtymbals to maximize the visibility of these shallow structures, with the ventral side towards the right, dorsal towards the left, anterior towards the top, and posterior towards the bottom.

**Additional file 8: **Correlation matrix. Pearson correlation coefficients between measured click rate (CR), microtymbal count (MT), projected tymbal surface area (TYSA), projected thoracic surface area (THSA), and the ratio of the TYSA:THSA (T2T) are given. MT and CLADE were found to be most strongly and positively correlated (*r* = 0.66). TYSA, and by extension T2T, were also found to have a positive relationship with CR (*r* = 0.13 and *r* = 0.12, respectively), though relatively weak compared to MT. THSA was found to correlate only with TYSA (*r* = 0.51).

**Additional file 9:** Monophyletic clade definitions. CLADE values used in this paper correspond to those defined by the node joining the two taxa listed and all its descendants. See Fig. 3 in [51] for comparison.

**Additional file 10:** Plot T2T against CR by CLADE. Some clades exhibit a positive relationship between T2T and CR (i.e., Eupseudosomoids, Callimorphoids, Phaegopteroids), others show little to no relation (i.e., Ctenuchoids, Euchaetioids), and the remaining were found to have a negative relationship (i.e., Euchromioids, Cisthenoids).

## Data Availability

The datasets generated and/or analyzed during the current study are available in the FigShare repositories: Raw Acoustic and Tymbal Measurements (10.6084/m9.figshare.9729857), R Scripts (10.6084/m9.figshare.9729860), Figures, Tables, and Supplementals in PDF format (10.6084/m9.figshare.9729869), Tables and Supplemental Tables in LaTeX Format (10.6084/m9.figshare.9729866). Audio files, SEM images, and/or voucher images of specimens generated in the current study are available from the corresponding author on reasonable request.
