# Supplementary material for: Characteristics of tiger moth (Erebidae: Arctiinae) anti-bat sounds can be predicted from tymbal morphology
Source: Front Zool. 2019 Dec 10;16:45. doi: 10.1186/s12983-019-0345-6 (PMC6902478; doi:10.1186/s12983-019-0345-6)
Supplement: Supplementary file 1 — Additional file 1. Acoustic and morphological measurements. The recorded maximum click rates (CR), microtymbal counts (MT), projected tymbal surface area (TYSA; mm2), projected thorax surface area (THSA; mm2), and ratio of TYSA to THSA (T2T) are given for each individual included in the study. The higher taxonomic grouping each belongs to (CLADE), sex, and voucher identification numbers (id) are also given for each specimen. Species identifications were left at “sp” when definitive species level identifications required examination of gentilic morphology. The “cf” designation was used to indicate a close external similarity to a given species, but a definitive identification could not be made due to small deviations in external morphology. [file 12983_2019_345_MOESM1_ESM.pdf]

index	id	Genus species	Sex	CLADE	CR	MT	TYSA	THSA	T2T
1	AZ13_0001	*Bertholdia trigona*	Male	Eupseudosomoid	1550	32	1.10	19.03	0.06
2	NC13_0074	*Haploa clymene*	Male	Callimorphoid	1170	32	1.11	21.98	0.05
3	YAN13_0151	*Bertholdia partita*	Male	Eupseudosomoid	1100	23	1.02	13.23	0.08
4	YAN13_0083	*Idalus pichesensis*	Male	Eupseudosomoid	1070	29	0.95	16.33	0.06
5	YAN13_0088	*Melese amastris*	Female	Eupseudosomoid	1010	24	0.94	15.20	0.06
6	YAN13_0097	*Melese intensa*	Male	Eupseudosomoid	1000	26	1.59	15.28	0.10
7	YAN13_0012	*Cosmosoma cf centralis*	Male	Euchromioid	930	45	0.83	8.49	0.10
8	AZ12_0076	*Carales arizonensis*	Male	Phaegopteroid	910	28	1.54	15.31	0.10
9	YAN13_0169	*Bertholdia ockendeni*	Male	Eupseudosomoid	840	24	1.15	17.87	0.06
10	YAN13_0173	*Elysius melaleuca*	Male	Phaegopteroid	760	35	1.12	20.67	0.05
11	YAN13_0163	*Bertholdia sp*	Male	Eupseudosomoid	720	21	1.03	15.63	0.07
12	NC13_0067	*Pyrrharctia isabella*	Female	Callimorphoid	610	28	1.96	30.07	0.07
13	YAN13_0079	*Ischnocampa cf discopuncta*	Male	Phaegopteroid	560	33	0.71	6.41	0.11
14	TX14_0065	*Euerythra phasma*	Male	Euchaetioid	550	32	0.93	15.44	0.06
15	YAN13_0150	*Pachydota rosenbergi*	Female	Phaegopteroid	430	14	1.81	31.00	0.06
16	AZ12_0009	*Pseudohemihyalea edwardsii*	Female	Phaegopteroid	380	20	1.48	34.82	0.04
17	YAN13_0006	*Ischnocampa nubilosa*	Male	Phaegopteroid	380	13	1.23	8.27	0.15
18	YAN13_0053	*Leucanopsis falacra*	Male	Phaegopteroid	380	19	1.13	24.41	0.05
19	YAN13_0119	*Phaegoptera decrepidoides*	Male	Phaegopteroid	380	11	1.02	14.92	0.07
20	NC12_0005	*Cycnia tenera*	Male	Euchaetioid	370	14	0.82	9.51	0.09
21	YAN13_0008	*Ischnocampa cf nubilosa*	Male	Phaegopteroid	370	11	1.15	6.89	0.17
22	YAN13_0041	*Ischnocampa cf obscurata*	Male	Phaegopteroid	370	17	1.46	8.97	0.16
23	YAN13_0045	*Phaio acquiguttata*	Male	Euchromioid	360	24	1.63	10.12	0.16
24	YAN13_0046	*Eucereon cf coeruleocaput*	Male	Ctenuchoid	340	33	1.03	13.50	0.08
25	YAN13_0058	*Praeamastus minerva minerva*	Female	Phaegopteroid	320	19	0.52	15.51	0.03
26	YAN13_0051	*Pelochyta gandolfii*	Male	Phaegopteroid	310	21	0.85	18.10	0.05
27	AZ13_0069	*Pygarctia roseicapitis*	Male	Euchaetioid	310	20	0.72	7.48	0.10
28	YAN13_0114	*Leucanopsis cf falacra*	Male	Phaegopteroid	300	19	0.45	8.37	0.05
29	YAN13_0121	*Ischnocampa cf hemihyalea*	Female	Phaegopteroid	300	12	0.87	7.55	0.12
30	YAN13_0110	*Pelochyta umbrata*	Male	Phaegopteroid	290	42	0.62	16.06	0.04
31	YAN13_0096	*Poecilosoma vespoides*	Male	Euchromioid	260	23	1.12	7.60	0.15
32	AZ12_0052	*Cisthene martini*	Male	Cisthenoid	230	12	0.33	3.92	0.08
33	YAN13_0061	*Praeamastus minerva watkinsi*	Male	Phaegopteroid	220	19	0.44	18.05	0.02
34	YAN13_0156	*Mesothen nomia*	Male	Euchromioid	210	20	1.37	8.99	0.15
35	YAN13_0161	*Correbia bricenoi*	Male	Ctenuchoid	210	12	1.42	12.97	0.11
36	YAN13_0020	*Eucereon coeruleocaput*	Male	Ctenuchoid	200	18	1.23	8.49	0.14
37	AZ12_0015	*Euchaetes antica*	Female	Euchaetioid	190	10	0.57	10.94	0.05
38	NC13_0071	*Euchaetes egle*	Male	Euchaetioid	180	18	0.69	8.61	0.08
39	YAN13_0135	*Eucereon rogersi*	Female	Ctenuchoid	180	21	1.95	20.05	0.10
40	YAN13_0165	*Leucanopsis bactris*	Male	Phaegopteroid	170	27	0.79	12.20	0.06
41	YAN13_0172	*Lophocampa cf endrolepia*	Male	Phaegopteroid	150	0	0.53	10.38	0.05
42	YAN13_0019	*Leucanopsis cf tabernilla*	Male	Phaegopteroid	130	12	0.87	15.51	0.06
43	AZ13_0003	*Ctenucha venosa*	Male	Ctenuchoid	110	6	2.54	20.47	0.12
44	YAN13_0004	*Amastus coccinator*	Male	Phaegopteroid	110	7	0.78	33.81	0.02
45	YAN13_0129	*Eucereon pseudocasca*	Female	Ctenuchoid	110	10	1.19	11.39	0.10
46	YAN13_0106	*Ischnocampa cf lugubris*	Male	Phaegopteroid	100	0	0.67	9.50	0.07
47	YAN13_0116	*Amastus ambrosia*	Male	Phaegopteroid	80	20	0.86	46.85	0.02
48	YAN13_0118	*Aemilia melanchra*	Male	Phaegopteroid	80	38	0.55	9.48	0.06
49	YAN13_0152	*Leucanopsis cf oruba*	Male	Phaegopteroid	70	19	0.73	14.44	0.05
50	YAN13_0074	*Aemilia rubriplaga*	Male	Phaegopteroid	60	0	0.69	11.06	0.06
51	YAN13_0133	*Lophocampa distincta*	Male	Phaegopteroid	50	0	0.37	11.94	0.03
52	YAN13_0134	*Praeamastus sp*	Female	Phaegopteroid	40	15	1.09	32.40	0.03
53	YAN13_0085	*Pseudohemihyalea vitripennis*	Male	Phaegopteroid	30	12	0.43	12.29	0.03
54	YAN13_0038	*Amastus erganoides*	Male	Phaegopteroid	20	14	0.67	25.42	0.03
55	YAN13_0057	*Opharus subflavus*	Male	Phaegopteroid	20	7	0.39	15.80	0.02
56	YAN13_0138	*Eucereon lineata*	Female	Ctenuchoid	10	0	1.82	18.79	0.10
57	AZ12_0002	*Eudesmia arida*	Male	Cisthenoid	0	0	0.44	5.18	0.08
58	MI17_0001	*Hypercompe scribonia*	Male	Callimorphoid	0	0	2.41	52.38	0.05
59	TX17_0001	*Hyphantria cunea*	Male	Callimorphoid	0	0	0.50	11.05	0.05
60	YAN13_0028	*Amastus rosenbergi*	Female	Phaegopteroid	0	15	0.62	23.22	0.03
61	YAN13_0034	*Palaeomolis palmeri*	Male	Callimorphoid	0	0	0.52	10.12	0.05
62	YAN13_0043	*Cisthene sp*	Male	Cisthenoid	0	15	0.61	3.92	0.16
63	YAN13_0070	*Palaeomolis rothschildi*	Male	Callimorphoid	0	0	0.80	15.43	0.05
64	YAN13_0139	*Hypercompe robusta*	Male	Callimorphoid	0	0	1.69	45.46	0.04
65	YAN13_0154	*Amaxia juvenis*	Male	Eupseudosomoid	0	0	0.64	11.35	0.06
66	YAN13_0157	*Elysius deceptura*	Male	Phaegopteroid	0	10	0.49	13.14	0.04
67	YAN13_0158	*Pseudischnocampa nervosa*	Male	Phaegopteroid	0	20	3.23	31.57	0.10
68	YAN13_0162	*Pseudepimolis syrissa*	Male	Eupseudosomoid	0	0	1.04	18.79	0.06
69	YAN13_0164	*Amaxia juvenis*	Male	Eupseudosomoid	0	0	0.87	15.00	0.06
70	YAN13_0167	*Holophaea endoleuca*	Female	Ctenuchoid	0	0	0.18	5.18	0.03
