# Supplementary material for: Characteristics of tiger moth (Erebidae: Arctiinae) anti-bat sounds can be predicted from tymbal morphology
Source: Front Zool. 2019 Dec 10;16:45. doi: 10.1186/s12983-019-0345-6 (PMC6902478; doi:10.1186/s12983-019-0345-6)
Supplement: Supplementary file 2 — Additional file 2: Descriptive statistics and distributions of CR, MT, and T2T. Individual data points from the 70 individuals included in our analyses are plotted, along with information about their distributions and summary statistics. [file 12983_2019_345_MOESM2_ESM.pdf]

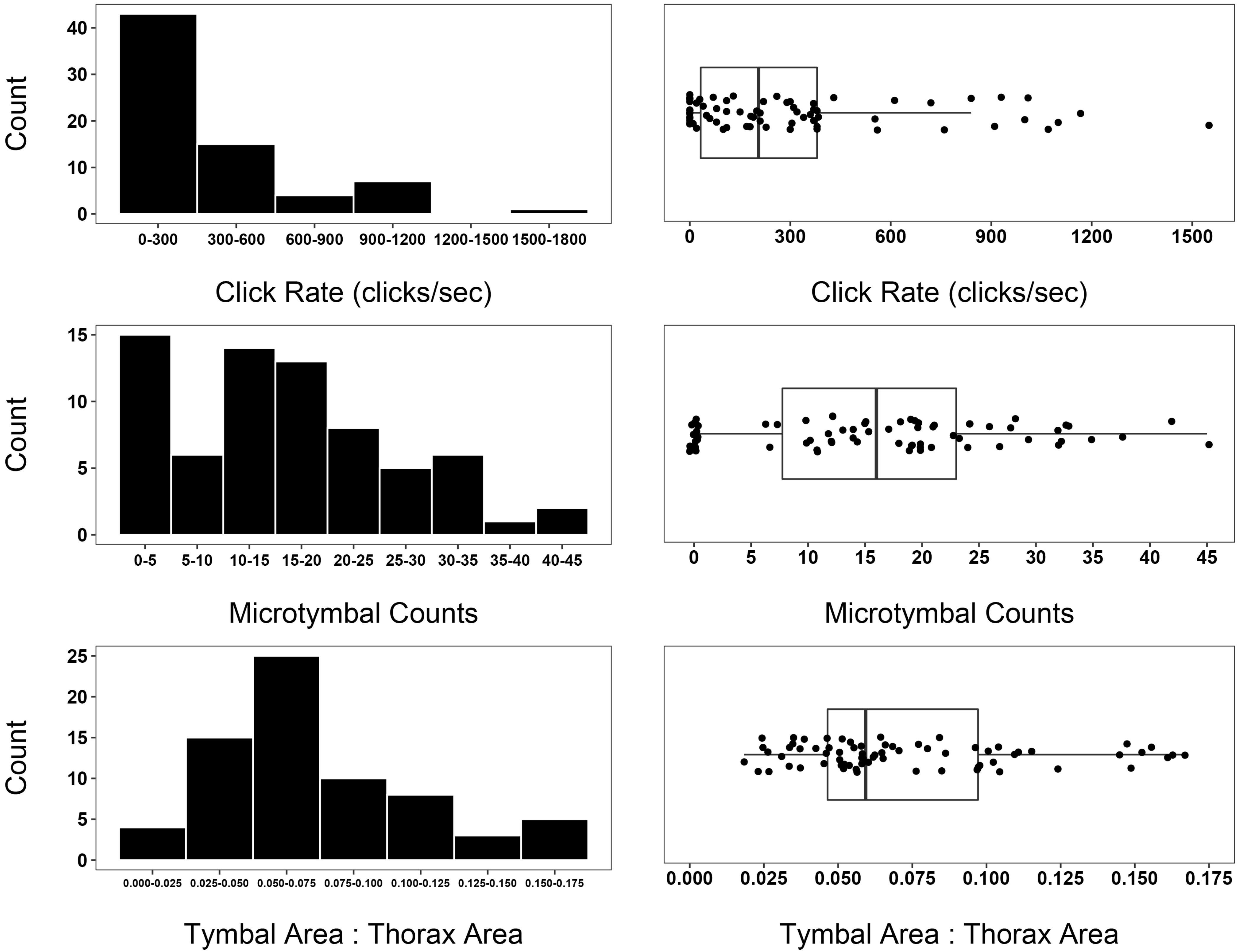

	n	Min	Mean	Median	Max	Std. Dev.	Std. Err.	Upper 95% CI	Lower 95% CI	Units
Click Rate	70	0.00	308.43	205.00	1550.00	350.65	82.14	390.57	226.29	clicks*sec^{-1}
Microtymbal Count	70	0.00	16.01	16.00	45.00	11.58	2.71	18.72	13.30	count
Tymbal:Thorax Area	70	0.02	0.07	0.06	0.17	0.04	0.01	0.08	0.06	mm^2
