# Supplementary material for: Characteristics of tiger moth (Erebidae: Arctiinae) anti-bat sounds can be predicted from tymbal morphology
Source: Front Zool. 2019 Dec 10;16:45. doi: 10.1186/s12983-019-0345-6 (PMC6902478; doi:10.1186/s12983-019-0345-6)
Supplement: Supplementary file 3 — Additional file 3: AICc Model Comparisons. The relative performance of all examined models are given with their Corrected Akaike’s Information Criterion (AICc), ordered from strongest likelihood to weakest likelihood. Models within a ∆ AICc of 2 relative to the most likely model (i.e., M11) are considered to be equally supported. [file 12983_2019_345_MOESM3_ESM.pdf]

Model	K	AICc	Delta AICc	AICc weight	log-Likelihood
M11: CR ~ MT + T2T + MT:CLADE	10	922.96	0.00	0.61	-449.61
M9: CR ~ MT + MT:CLADE	9	923.92	0.96	0.38	-451.46
M15: CR ~ MT + T2T + CLADE + MT:CLADE	16	932.54	9.59	0.01	-445.14
M13: CR ~ MT + CLADE + MT:CLADE	15	937.28	14.32	0.00	-449.20
M17: CR ~ MT + T2T + CLADE + MT:CLADE + T2T:CLADE	22	945.14	22.18	0.00	-439.80
M18: CR ~ MT + T2T + CLADE + MT:CLADE + T2T:CLADE + MT:T2T	23	949.05	26.09	0.00	-439.52
M12: CR ~ MT + T2T + T2T:CLADE	10	961.45	38.49	0.00	-468.86
M7: CR ~ MT + CLADE	9	963.98	41.02	0.00	-471.49
M16: CR ~ MT + T2T + CLADE + T2T:CLADE	16	975.89	52.94	0.00	-466.81
M1: CR ~ MT	3	984.87	61.91	0.00	-489.25
M3: CR ~ MT + T2T	4	986.98	64.03	0.00	-489.18
M4: CR ~ MT + MT:T2T	4	987.00	64.05	0.00	-489.19
M6: CR ~ MT + T2T + MT:T2T	5	989.31	66.35	0.00	-489.18
M5: CR ~ T2T + MT:T2T	4	993.66	70.71	0.00	-492.52
M10: CR ~ T2T + T2T:CLADE	9	1010.76	87.80	0.00	-494.88
M8: CR ~ T2T + CLADE	9	1012.96	90.01	0.00	-495.98
M14: CR ~ T2T + CLADE + T2T:CLADE	15	1021.08	98.12	0.00	-491.10
M0: Intercept Only (Null)	2	1022.22	99.26	0.00	-509.02
M2: CR ~ T2T	3	1023.30	100.34	0.00	-508.47
