# Supplementary figures and images for: Characteristics of tiger moth (Erebidae: Arctiinae) anti-bat sounds can be predicted from tymbal morphology
Source: Front Zool. 2019 Dec 10;16:45. doi: 10.1186/s12983-019-0345-6 (PMC6902478; doi:10.1186/s12983-019-0345-6)

Click Rate (clicks/sec)

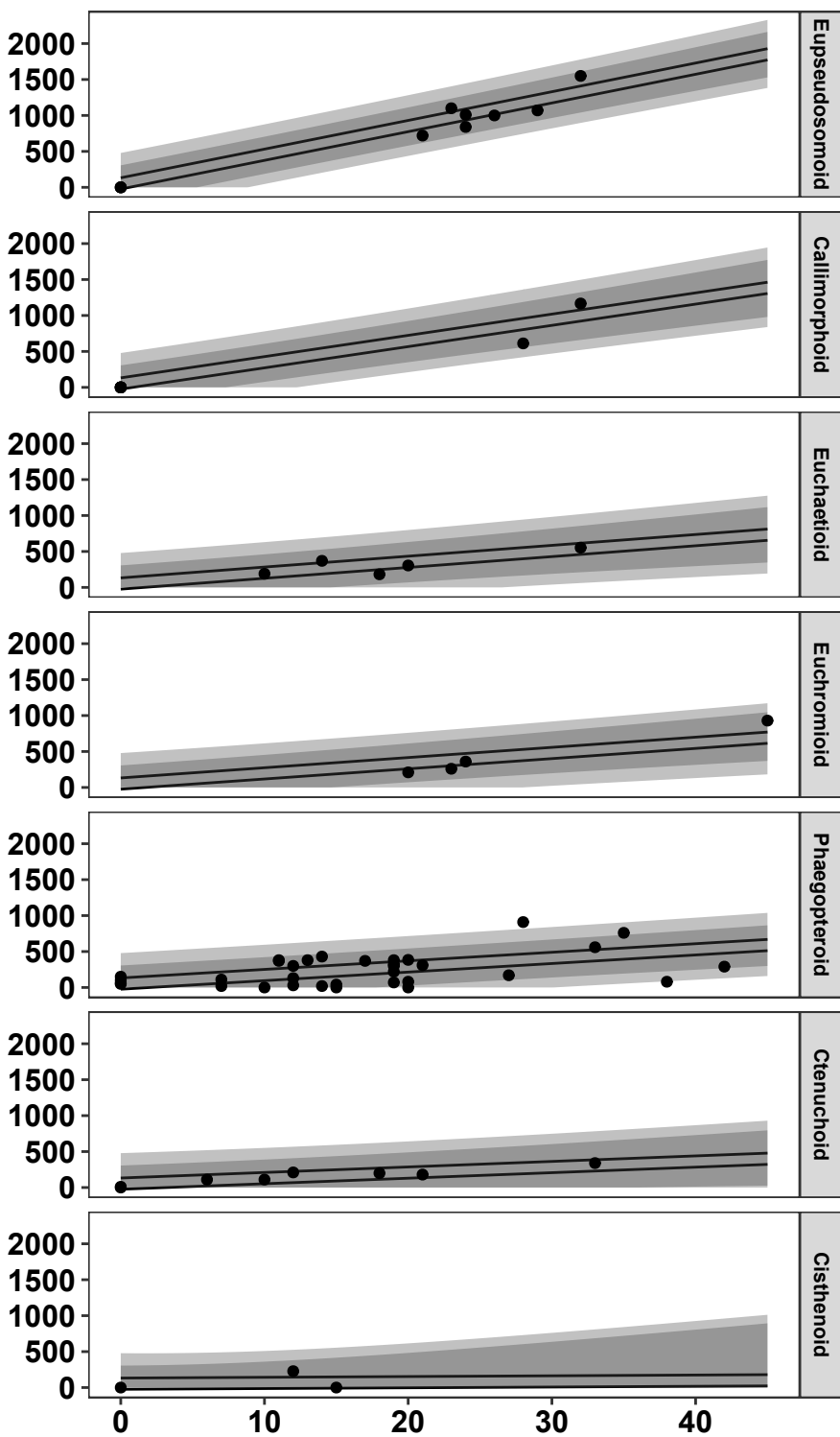

Microtymbal Count

Supplement: Supplementary file 4 — Additional file 4: Model 11 with 95% Prediction Intervals. For a given MT, CLADE and T2T, CR is expected to fall within these intervals in 95% of cases. T2T shifts this prediction interval up or down depending on its value. In order to present the prediction intervals for Model 11 in a 2D graphic, we plotted two ribbons which represent the minimum (1.8%; dark grey) and maximum (16.7%; light grey) T2T values observed in this study. This shows the extent that the prediction interval could be expected to shift if two individuals within the same CLADE had the same MT, but extremely different T2T. CR which were predicted to be negative values (e.g., − 100 clicks/second) were set to 0 clicks/sec because negative rates would not be biologically meaningful. [file 12983_2019_345_MOESM4_ESM.pdf]

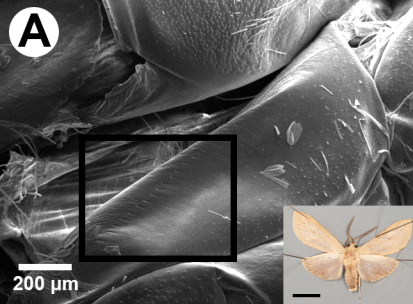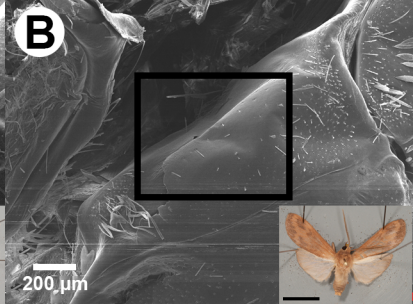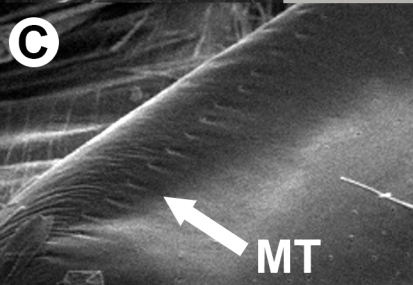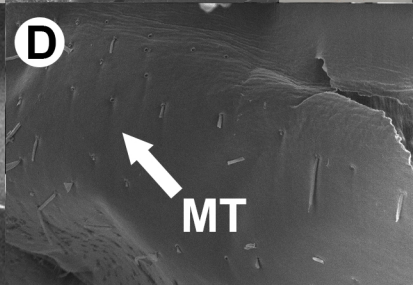

Supplement: Supplementary file 7 — Additional file 7: Comparison of normal and putatively vestigial microtymbal morphology. Exemplar microtymbal (MT) morphology of two species from closely related genera within the same CLADE (Phaegopteroid). A) tymbal of Leucanopsis cf falacra (index: 28; id: YAN13_0114), scale bar = 200 μm; inset: dorsal view of specimen, scale bar = 1 cm. B) tymbal of Elysius deceptura (index: 66; id: YAN13_0157), scale bar = 200 μm; inset: dorsal view of specimen, scale bar = 1 cm. C) represents a normal state (MT = 19; CR = 300) with regularly spaced, deep, and well-aligned microtymbals. D) represents a putatively vestigial state (MT = 10; CR = 0) with irregularly spaced, shallow, and misaligned microtymbals. A-C are oriented with the anterior side towards the left, posterior towards the right, dorsal towards the top, and ventral towards the bottom. D is oriented looking down the row of microtymbals to maximize the visibility of these shallow structures, with the ventral side towards the right, dorsal towards the left, anterior towards the top, and posterior towards the bottom. [file 12983_2019_345_MOESM7_ESM.pdf]

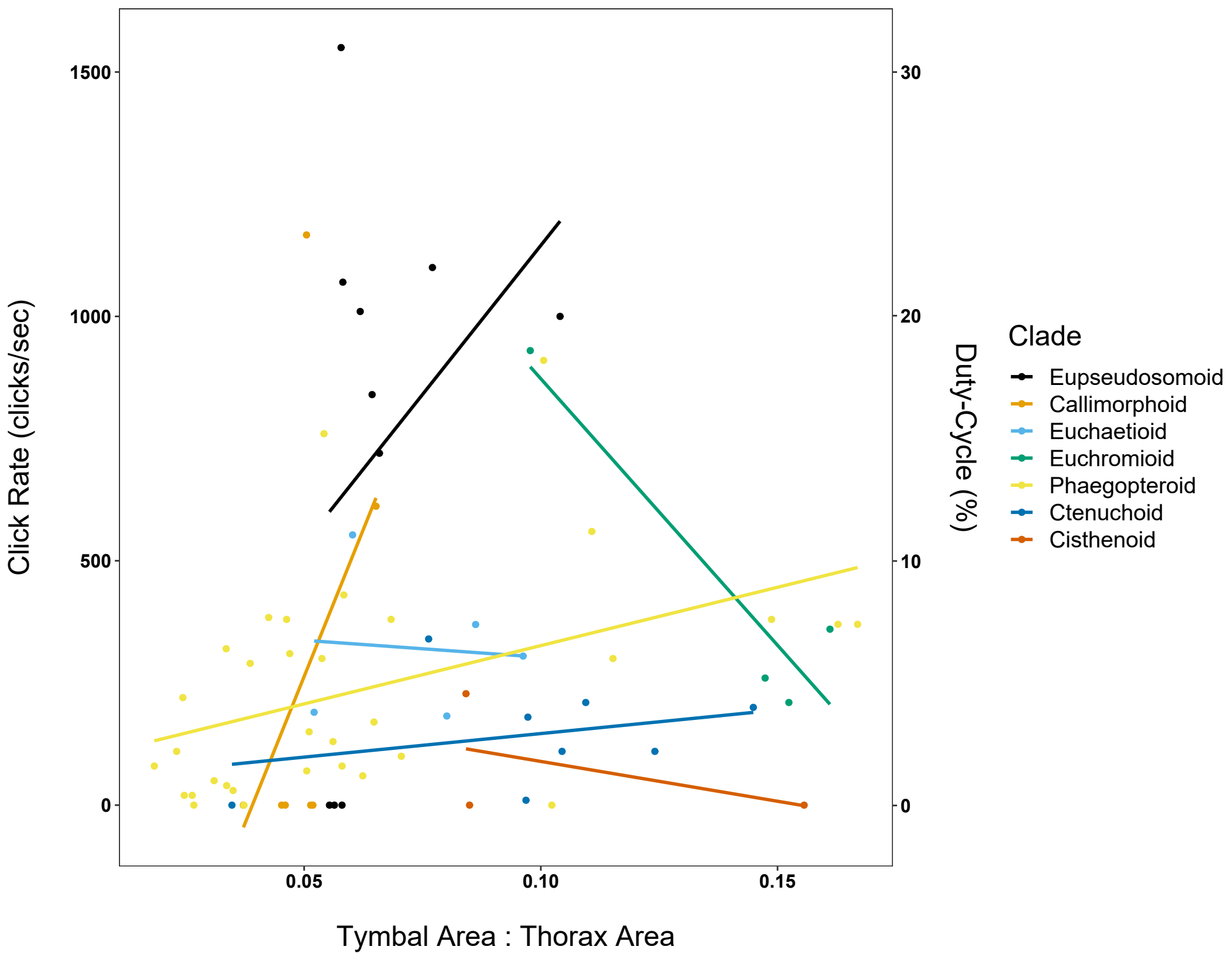

Supplement: Supplementary file 10 — Additional file 10: Plot T2T against CR by CLADE. Some clades exhibit a positive relationship between T2T and CR (i.e., Eupseudosomoids, Callimorphoids, Phaegopteroids), others show little to no relation (i.e., Ctenuchoids, Euchaetioids), and the remaining were found to have a negative relationship (i.e., Euchromioids, Cisthenoids). [file 12983_2019_345_MOESM10_ESM.pdf]
