# Supplementary material for: Characteristics of tiger moth (Erebidae: Arctiinae) anti-bat sounds can be predicted from tymbal morphology
Source: Front Zool. 2019 Dec 10;16:45. doi: 10.1186/s12983-019-0345-6 (PMC6902478; doi:10.1186/s12983-019-0345-6)
Supplement: Supplementary file 5 — Additional file 5: Contrast matrix for Model 9. The modeled relationship between click rate and microtymbal count clusters into 3 significantly different groups: Eupseudosomoids, Callimorphoids, and all other clades. Apart from the Cisthenoid clade, all clades were found to have a significantly positive relationship between microtymbal count and click rate. The significance and the magnitude of the slope differences are relative to the unlisted clade for each contrast column. Overall, Model 9 accounts for CR well (Adj. R2 = 0.79), while only requiring 2 factors to be measured (i.e., MT and CLADE). [file 12983_2019_345_MOESM5_ESM.pdf]

	Contrast 1	Contrast 2	Contrast 3	Contrast 4	Contrast 5	Contrast 6	Contrast 7
(Intercept)	25.14	25.14	25.14	25.14	25.14	25.14	25.14
	(34.04)	(34.04)	(34.04)	(34.04)	(34.04)	(34.04)	(34.04)
MT	40.09^{***}	29.29^{***}	15.55^{***}	16.39^{***}	11.83^{***}	9.54^{*}	5.58
	(2.71)	(3.98)	(3.92)	(2.94)	(2.07)	(3.86)	(8.82)
MT:Eupseudosomoid		10.81^{*}	24.54^{***}	23.70^{***}	28.26^{***}	30.55^{***}	34.52^{***}
		(4.51)	(4.32)	(3.63)	(2.79)	(4.26)	(8.87)
MT:Callimorphoid	-10.81^{*}		13.73^{*}	12.90^{**}	17.46^{***}	19.74^{***}	23.71^{*}
	(4.51)		(5.26)	(4.70)	(4.10)	(5.21)	(9.38)
MT:Euchaetioid	-24.54^{***}	-13.73^{*}		-0.84	3.72	6.01	9.98
	(4.32)	(5.26)		(4.54)	(3.87)	(5.03)	(9.24)
MT:Euchromioid	-23.70^{***}	-12.90^{**}	0.84		4.56	6.85	10.81
	(3.63)	(4.70)	(4.54)		(3.12)	(4.49)	(9.00)
MT:Phaegopteroid	-28.26^{***}	-17.46^{***}	-3.72	-4.56		2.29	6.25
	(2.79)	(4.10)	(3.87)	(3.12)		(3.80)	(8.64)
MT:Ctenuchoid	-30.55^{***}	-19.74^{***}	-6.01	-6.85	-2.29		3.97
	(4.26)	(5.21)	(5.03)	(4.49)	(3.80)		(9.20)
MT:Cisthenoid	-34.52^{***}	-23.71^{*}	-9.98	-10.81	-6.25	-3.97	
	(8.87)	(9.38)	(9.24)	(9.00)	(8.64)	(9.20)	
R^2	0.81	0.81	0.81	0.81	0.81	0.81	0.81
Adj. R^2	0.79	0.79	0.79	0.79	0.79	0.79	0.79
Num. obs.	70	70	70	70	70	70	70
RMSE	162.49	162.49	162.49	162.49	162.49	162.49	162.49

$^{***}p < 0.001$, $^{**}p < 0.01$, $^{*}p < 0.05$
