# Supplementary material for: Characteristics of tiger moth (Erebidae: Arctiinae) anti-bat sounds can be predicted from tymbal morphology
Source: Front Zool. 2019 Dec 10;16:45. doi: 10.1186/s12983-019-0345-6 (PMC6902478; doi:10.1186/s12983-019-0345-6)
Supplement: Supplementary file 6 — Additional file 6: Contrast matrix for Model 11. The modeled relationship between click rate and microtymbal count clusters into 3 significantly different groups: Eupseudosomoids, Callimorphoids, and all other clades. Apart from the Cisthenoid clade, all clades were found to have a significantly positive relationship between microtymbal count and click rate. The significance and the magnitude of the slope differences are relative to the unlisted clade for each contrast column. Overall, Model 11 accounts for CR as well as Model 9 (both Adj. R2 = 0.79), while requiring 3 factors to be measured (i.e., MT, CLADE, and T2T). [file 12983_2019_345_MOESM6_ESM.pdf]

	Contrast 1	Contrast 2	Contrast 3	Contrast 4	Contrast 5	Contrast 6	Contrast 7
(Intercept)	−43.47	−43.47	−43.47	−43.47	−43.47	−43.47	−43.47
	(49.93)	(49.93)	(49.93)	(49.93)	(49.93)	(49.93)	(49.93)
MT	39.92***	29.56***	15.09***	14.18***	11.92***	7.69	1.05
	(2.66)	(3.91)	(3.85)	(3.12)	(2.03)	(3.92)	(8.99)
T2T	1053.52	1053.52	1053.52	1053.52	1053.52	1053.52	1053.52
	(569.90)	(569.90)	(569.90)	(569.90)	(569.90)	(569.90)	(569.90)
MT:Eupseudosomoid		10.36*	24.83***	25.74***	28.00***	32.23***	38.87***
		(4.43)	(4.24)	(3.73)	(2.74)	(4.27)	(9.01)
MT:Callimorphoid	−10.36*		14.47**	15.38**	17.64***	21.87***	28.51**
	(4.43)		(5.18)	(4.80)	(4.02)	(5.24)	(9.56)
MT:Euchaetioid	−24.83***	−14.47**		0.91	3.17	7.40	14.04
	(4.24)	(5.18)		(4.56)	(3.81)	(4.99)	(9.32)
MT:Euchromioid	−25.74***	−15.38**	−0.91		2.26	6.49	13.13
	(3.73)	(4.80)	(4.56)		(3.30)	(4.40)	(8.92)
MT:Phaegopteroid	−28.00***	−17.64***	−3.17	−2.26		4.23	10.87
	(2.74)	(4.02)	(3.81)	(3.30)		(3.88)	(8.84)
MT:Ctenuchoid	−32.23***	−21.87***	−7.40	−6.49	−4.23		6.64
	(4.27)	(5.24)	(4.99)	(4.40)	(3.88)		(9.15)
MT:Cisthenoid	−38.87***	−28.51**	−14.04	−13.13	−10.87	−6.64	
	(9.01)	(9.56)	(9.32)	(8.92)	(8.84)	(9.15)	
R^2	0.82	0.82	0.82	0.82	0.82	0.82	0.82
Adj. R^2	0.79	0.79	0.79	0.79	0.79	0.79	0.79
Num. obs.	70	70	70	70	70	70	70
RMSE	159.41	159.41	159.41	159.41	159.41	159.41	159.41

$^{***}p < 0.001$, $^{**}p < 0.01$, $^{*}p < 0.05$
