# Supplementary material for: Characteristics of tiger moth (Erebidae: Arctiinae) anti-bat sounds can be predicted from tymbal morphology
Source: Front Zool. 2019 Dec 10;16:45. doi: 10.1186/s12983-019-0345-6 (PMC6902478; doi:10.1186/s12983-019-0345-6)
Supplement: Supplementary file 8 — Additional file 8: Correlation matrix. Pearson correlation coefficients between measured click rate (CR), microtymbal count (MT), projected tymbal surface area (TYSA), projected thoracic surface area (THSA), and the ratio of the TYSA:THSA (T2T) are given. MT and CLADE were found to be most strongly and positively correlated (r = 0.66). TYSA, and by extension T2T, were also found to have a positive relationship with CR (r = 0.13 and r = 0.12, respectively), though relatively weak compared to MT. THSA was found to correlate only with TYSA (r = 0.51). [file 12983_2019_345_MOESM8_ESM.pdf]

	CR	MT	TYSA	THSA	T2T
CR	1	-	-	-	-
MT	0.66	1	-	-	-
TYSA	0.13	0.09	1	-	-
THSA	-0.05	-0.04	0.51	1	-
T2T	0.12	0.14	0.38	-0.45	1
