# Supplementary material for: Characteristics of tiger moth (Erebidae: Arctiinae) anti-bat sounds can be predicted from tymbal morphology
Source: Front Zool. 2019 Dec 10;16:45. doi: 10.1186/s12983-019-0345-6 (PMC6902478; doi:10.1186/s12983-019-0345-6)
Supplement: Supplementary file 9 — Additional file 9: Monophyletic clade definitions. CLADE values used in this paper correspond to those defined by the node joining the two taxa listed and all its descendants. See Fig. 3 in [51] for comparison. [file 12983_2019_345_MOESM9_ESM.pdf]

Clade Name	Branch 1	Branch 2
Eupseudosomoid	*Cissura decora*	*Ordishia rutilus*
Callimorphoid	*Utetheisa lotrix*	*Virbia fragilis*
Euchaetioid	*Pagara simplex*	*Agaraea semivitrea*
Euchromioid	*Macrocneme sp.*	*Dycladia lucetius*
Phaegopteroid	*Anaxita sp. 1*	*Pachydota affinis*
Ctenuchoid	*Neotrichura nigripes*	*Ctenucha virginica*
Cisthenoid	*Praepiella sesapina*	*Ardonea tenebrosa*
